# Surgeon General’s Call to Action to Prevent Skin Cancer

**Published:** 2014-08-01

On July 29, the Office of the Surgeon General released its Call to Action to Prevent Skin Cancer (http://www.surgeongeneral.gov/library/calls/prevent-skin-cancer/index.html). Skin cancer is the most frequently diagnosed cancer in the United States, with nearly 5 million persons treated each year, at an estimated cost of $8.1 billion dollars (1–4). Melanoma is responsible for most skin cancer–related deaths, causing approximately 9,000 deaths annually (5).

Most cases of skin cancer are preventable, yet skin cancer rates have continued to increase in the United States during the past 35 years (6). Despite efforts to increase use of sun protection, approximately one third of U.S. residents reported being sunburned in the past year (7). In addition, indoor tanning greatly increases the risk for developing skin cancer, yet one out of every three non-Hispanic white women aged 16–25 years engages in indoor tanning each year (8).

The purpose of the Surgeon General’s Call to Action is to increase awareness of skin cancer as a national public health priority, as well as to engage stakeholders across the nation, including policy makers; members of the business, health care, and education sectors; community, nonprofit, and faith-based organizations; and individuals and families, all with concrete strategies to reduce the risk for skin cancer.
